# Silicon Supply Improves the Rhizodeposition and Transfer of Nitrogen from *Trifolium incarnatum* L. to *Brassica napus* L.

**DOI:** 10.3390/plants14081246

**Published:** 2025-04-19

**Authors:** Raphaël Coquerel, Mustapha Arkoun, Philippe Laîné, Philippe Etienne

**Affiliations:** 1 Université de Caen Normandie, INRAE, UMR 950 EVA, SF Normandie Végétal (FED4277), 14000 Caen, France; raphael.coquerel@gmail.com (R.C.); philippe.laine@unicaen.fr (P.L.); 2 Laboratoire de Nutrition Végétale, Centre Mondial d’Innovation-Groupe Roullier, 35400 Saint-Malo, France; mustapha.arkoun@roullier.com

**Keywords:** agro-ecological transition, crop associations, nitrogen use efficiency, ^15^N labeling, split-root system

## Abstract

The association of legumes with other non-legume plants, such as *Brassica napus* L., has been reported as an agro-ecological alternative for reducing the nitrogen (N) inputs required for *B. napus* growth, thanks in particular to the transfer of N compounds from the legume to *B. napus*. Moreover, recent studies have evidenced that silicon (Si) supply can improve either N uptake by *B. napus* or the dinitrogen fixation capacity of *T. incarnatum*. However, the effect of Si supply on the N nutrition of both *B. napus* and *T. incarnatum*, especially when growing in association, has not been assessed so far. The aim of this study was to assess the effect of Si supply on the growth of *B. napus* and *T. incarnatum* cultivated in association by focusing particularly on N rhizodeposition by *T. incarnatum* and its transfer to *B. napus*. The experiment was performed for 10 weeks under a split-root system combined with an ^15^N labeling method. The results showed that the Si supply increased the amount of rhizo-deposited N by *T. incarnatum* by over 40% and enhanced its transfer to *B. napus*. The transferred N was allocated mainly to pods (17%), as their biomass increased under Si supply. For the first time, this study demonstrates that the association with legume plants together with the Si supply could be an effective approach to improve the agro-ecological balance of *B. napus*.

## 1. Introduction

Nitrogen (N) fertilization is widely used to ensure the growth, yield, and quality of the harvested products of many field crops [1,2]. However, the intensive use of N fertilizers over more than six decades has led to undesirable economic and environmental consequences [3]. For example, the industrial synthesis of N fertilizers, which consumes a lot of fossil fuels, releases large quantities of greenhouse gases into the atmosphere that are harmful to human health [4]. In addition, several studies have reported that the high doses of N used to fertilize field crops can cause soil acidification [5] and lead to groundwater contamination [6] and/or greenhouse gas emissions [7] by N leaching and/or volatilization. Due to these negative impacts, it is urgent to explore an alternative approach to sustainable cropping practices through the minimization of exogenous N supply without compromising the agronomic performance of field crops.

To achieve this objective, the association of legume plants, suitable for fixing atmospheric dinitrogen (N_2_), with non-legume plants has been widely reported to be one of the most sustainable ways of introducing N into agroecosystems [8,9,10]. Even though several decades of research into crop associations have mainly focused on corn, soybeans, and wheat [11], more recent attention has been paid to *Brassica napus* L., which is another crop that requires high doses of N fertilizer [12]. In these associations, legumes fix N_2_ and rhizodeposit N-compounds into the soil that can then be taken up by the non-fixing companion plant [9,13]. As previously reported, the extent of N rhizodeposition resulting from the mineralization of litter, root necromass, and/or root exudation of N-compounds is different between legume species, especially due to the variation in their N_2_-fixation and exudation capacities [14]. For example, a previous study comparing different legume species (*Trifolium incarnatum* L., *Vicia sativa* L., and *Lupinus albus* L.) cultivated in association with *B. napus* L. reported that *T. incarnatum* L. and *L. albus* L. have much higher N_2_ fixation and N exudation capacities than *V. sativa* L. [15]. The same authors showed that, irrespective of the legume species, the N_2_-fixing capacity of the legumes cultivated in association was always higher than the monocrops. This result is explained by the high N uptake capacity of *B. napus* L., which leads to N impoverishment in the soil solution, thus promoting nodulation and N_2_ fixation capacity in legumes [15,16]. Using ^15^N labeling, these studies have also shown that the proportion of N transferred from legumes to *B. napus* L. accounts for over 3% of the total N in *B. napus* L. [17]. Furthermore, field experiments have revealed that the association of *B. napus* L. and legumes could also decrease weed growth and insect pest pressure and improve N and sulfur use efficiency and, as a result, the growth of *B. napus* L. when compared to *B. napus* L. grown in monoculture [12,18,19].

Although these crop associations appear to be an effective cropping practice for increasing N use efficiency and reducing inputs in field crops, other strategies are also worth considering. Among them, silicon (Si) fertilization is recognized as a relevant practice for improving N use efficiency and agronomic performance in many crops, such as *Zea mays* L., *Oryza sativa* L., and *B. napus* L. [20,21]. Indeed, numerous studies have indicated that Si supply improves N nutrition by enhancing N uptake, assimilation, and/or remobilization in many plant species [22,23,24,25]. For example, in *B. napus* L. grown under N deprivation, Si supply leads to an increase in the expression of genes encoding root nitrate transporters (*BnaNRT1.1* and *BnaNRT1.2*), which allows better N uptake when nitrate is resupplied [23]. Under field conditions, an Si supply enhances the agronomic performance of *B. napus* L. by improving the usage of N fertilizer [26]. This result suggests that Si fertilization could allow a reduction in N inputs without altering *B. napus* L. growth and yield. In the same way, numerous studies have demonstrated that Si supply improves growth and N_2_ fixation capacity in many legume species [20,27]. For example, recent studies have reported that Si supply increases the number and size of nodules as well as their nitrogenase content in *T. incarnatum* L. cultivated under low sulfur or N availability. All of these factors, enhanced by Si, lead to an elevation in the N_2_ fixation capacity and growth of *T. incarnatum* L. [28,29]. In the literature, the positive effects of Si fertilization on N use efficiency are only observed in plants grown in monoculture and never in crop associations.

The aim of this study was to assess whether Si supply is able to improve the functioning of *B. napus* L. cultivated in association with *T. incarnatum* L. More precisely, the effects of Si supply on the amount of N rhizodeposited from *T. incarnatum* L. and its transfer and allocation to *B. napus* L. were examined.

## 2. Results

### 2.1. Effect of Si Supply on Biomass and Total N Amount in B. napus L. and T. incarnatum L.

**Figure 1:**
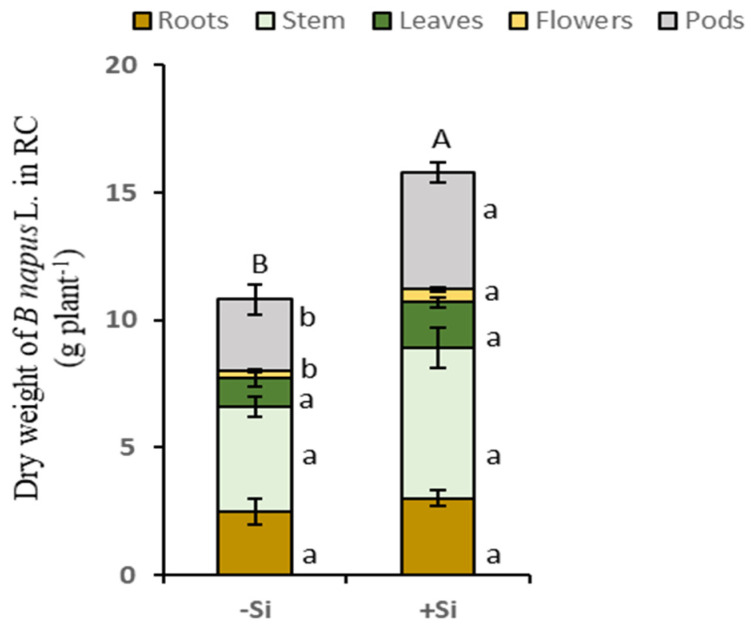
Dry weight of *Brassica napus* L. plants supplied with (+Si; 1.7 mM) or without silicon (−Si) and cultivated in soil (RC) for 10 weeks. Values correspond to the mean ± SE for n = 3. For each organ and whole-plant biomasses; data obtained from +Si plants were compared to −Si plants (control) using Student’s *t*-test. Different lowercase and uppercase letters indicate significant differences (*p* < 0.05) between organs and whole plants, respectively.

After 10 weeks, Si supply led to a significant increase (*p* < 0.05) in the total biomass of *B. napus* L. plants, reaching around 17 g DW plant^−1^ vs. 12 g DW g^−1^ in −Si plants (Figure 1). This increase in *B. napus* L. plant biomass is mainly explained by the higher biomass of pods and flowers, which increased by 1.6-fold and 2-fold, respectively. In *T. incarnatum* L., the addition of Si to the RC had no significant impact on total biomass, shoot biomass, or root biomass in RC or DC (Table 1). In *B. napus* L., the Si supply significantly (*p* < 0.05) increased the total N amount in the whole plant, leaves, pods, and flowers. In addition, Si supply led to an increase in the ^15^N isotopic excess in pods (from 1 to 5.9 atom% in −Si and +Si plants, respectively) and whole plants (from 1.4 to 4.1 atom% for −Si and +Si plants, respectively). In contrast, in *T*. *incarnatum* L., the total N amount and the ^15^N isotopic excess in whole plants and in their different compartments were similar, irrespective of the Si treatment (Table 2).

### 2.2. Effect of Si Supply on N Rhizodeposition from T. incarnatum L. and N Transfer to B. napus L.

An Si supply led to a significant increase (*p* < 0.05) in the amount of N rhizodeposited from *T. incarnatum* L. to the RC, with values increasing from 70.9 ± 7.6 to 99.8 ± 7.3 mg. In addition, the Si supply increased the N transfer (%), i.e., the proportion of *T. incarnatum* L. N transferred to *B. napus* L. (from 0.42 to 1.6%). This resulted in a significant increase (*p* < 0.05) in the amount of N transferred (by 4-fold) from *T. incarnatum* L. to *B. napus* L., which rose from 5.7 to 23.3 mg in −Si and +Si plants, respectively. Finally, the supply of Si resulted in an increase in the proportion of N derived from the transfer (%Ndft) in *B. napus* L., which reached 12.2 vs. 4.2% in +Si and −Si plants, respectively (Table 3).

### 2.3. Si Effect on the Distribution of N Derived from T. incarnatum L. in the Receiver Compartment

**Figure 2:**
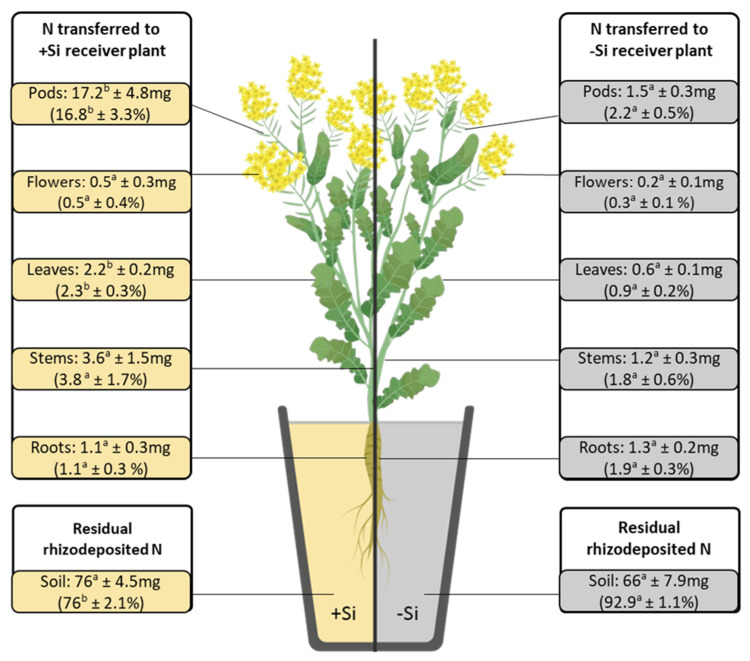
Distribution of total rhizodeposited N from *Trifolium incarnatum* L. (donor plant) to the receiver compartment (RC). The N amount transferred to the receiver plant and the residual N amount rhizodeposited from the donor plant to soil (RC) supplied with silicon (+Si; 1.7 mM; gold boxes) or without silicon (−Si; 0 mM; grey boxes) for 10 weeks. Values in percent indicated in brackets correspond to the proportion of rhizodeposited N remaining in soil or allocated in each *B. napus* compartment. Values correspond to the mean ± SE for n = 3. Data obtained from +Si plants were compared to −Si plants (control) using Student’s *t*-test and different letters indicate significant differences (*p* < 0.05) between the control (−Si) and +Si plants.

The distribution of rhizodeposited N from *T. incarnatum* L. in the receiver compartment (RC), i.e., in soil and the *B. napus* L. plant, is summarized in Figure 2. In RC, the amount of total N rhizodeposited from *T. incarnatum* L. increased from 70.9 ± 7.6 to 99.8 ± 7.3 mg in −Si RC and +Si RC, respectively (Table 3). In soil, despite the total rhizodeposited N amount, which was higher in +Si RC than −Si RC, the supply of Si did not lead to any change in the amount of residual rhizodeposited N (around 70 mg, irrespective of the Si supply, which could correspond to the amount of nitrogen immobilized by colloids and/or soil microorganisms; Table 3). Consequently, the proportion of residual rhizodeposited N was significantly decreased from around 90% to 75% in RC when Si was supplied (Figure 2). This result indicates that Si supply enhanced the rhizodeposited N uptake by *B. napus* L. and caused an increase in the amount of N transferred, as shown in Table 3. As presented in Figure 2, Si has no significant effect on the distribution of the N transferred within the roots, stems, and flowers because the amounts of transferred N (and the proportion of rhizodeposited N) were similar in these different compartments of *B. napus* L., regardless of the Si supply. In contrast, supplying Si increased the amount of N transferred to leaves (from 0.6 to 2.2 mg N) and especially to pods (from 1.5 to 17.2 mg N) in *B. napus* L. These results are due to a significant increase in the allocation of rhizodeposited N in these two organs. Indeed, the proportion of rhizodeposited N transferred in *B. napus* L. plants fed with Si reached 2.3% (vs. 0.9% in −Si plants) in leaves and 16.8% (*vs.* only 2.2% in −Si plants) in pods (Figure 2).

**Table 1:** Dry weight of *Trifolium incarnatum* L. (donor plant) for 10 weeks. The Si was only supplied (+Si; 1.7 mM) in the receiver (RC) and not supplied (−Si) in the donor compartment (DC). Roots in RC and DC indicate roots in the receiver compartment (soil) and the donor compartment (hydroponic), respectively (for details, see Figure 3). Values are mean ± SE for n = 3. Data obtained from +Si plants were compared to −Si plants (control) using Student’s *t*-test (*p* < 0.05). Different letters indicate significant differences (*p* < 0.05) between control (−Si) and +Si plants.

	Dry Weight (g Plant^−1^)			
	Roots		Shoots	Whole Plant
	in RC	in DC		
**−** **Si**	2.6^a^ ± 0.6	4.2^a^ ± 0.4	43.4^a^ ± 4.2	50.2^a^ ± 5.8
**+Si**	3.1^a^ ± 0.7	4.6^a^ ± 0.5	38.7^a^ ± 0.5	46.4^a^ ± 0.7

**Table 2:** Total N amounts and ^15^N excess of *Trifolium incarnatum* L. (donor plant) and *Brassica napus* L. (receiver plant) supplied with (+Si; 1.7 mM) or without silicon (−Si) for 10 weeks. The Si is only supplied (+Si) or not (−Si) in the receiver compartment (RC) and nutrient solution containing ^15^N labeling is only supplied in donor compartment (DC). For *T. incarnatum* L.); roots in RC and DC indicate roots in the receiver compartment (soil) and the donor compartment (hydroponic), respectively (for details, see Figure 3). Values correspond to mean ± SE for n = 3. Data obtained from +Si plants were compared to −Si plants (control) using Student’s *t*-test. For a given plant compartment or whole plant, different letters indicate significant differences (*p* < 0.05) between control (−Si) and +Si plants.

		*Brassica napus* L.						*Trifolium incarnatum* L.			
		Roots	Stems	Leaves	Flowers	Pods	Whole Plant	Roots		Shoots	Whole Plant
								in RC	in DC		
**N amount (mg plant^−1^)**	**−** **Si**	18^a^ ± 3	27^a^ ± 1	20^a^ ± 6	3^a^ ± 6	53^a^ ± 7	122^a^ ± 7	64^a^ ± 12	116^a^ ± 13	1227^a^ ± 74	1408^a^ ± 96
	**+Si**	20^a^ ± 3	42.6^a^ ± 6	40^b^ ± 4	6^b^ ± 0	78^b^ ± 1	185^b^ ± 12	96^a^ ± 17	133^a^ ± 5	1215^a^ ± 200	1445^a^ ± 207
**^15^** **N excess (atom %)**	**−** **Si**	3.2^a^ ± 1.1	1.5^a^ ± 0.9	2.2^a^ ± 1.3	1.8^a^ ± 0.4	1.0^a^ ± 0.2	1.4^a^ ± 0.2	19.9^a^ ± 6.3	53.9^a^ ± 2.0	32.2^a^ ± 4.7	33.4^a^ ± 4.5
	**+Si**	2^a^ ± 0.8	3.2^a^ ± 1.6	2.3^a^ ± 0.4	2.7^a^ ± 1.9	5.9^b^ ± 0.6	4.1^b^ ± 0.7	13.9^a^ ± 1.3	59.9^a^ ± 3.7	31.4^a^ ± 2.8	32.6^a^ ± 2.7

**Table 3:** Nitrogen (N) rhizodeposited by *Trifolium incarnatum* L. (donor plant) in the receiver compartment (RC) and transferred to *Brassica napus* L. (receiver plant). The Si is only supplied (+Si) or not (−Si) in the receiver compartment (RC), and the nutrient solution containing ^15^N labeling is supplied only in the donor compartment (DC). N rhizodeposited corresponds to the total amount of N rhizodeposited from *T. incarnatum* L. (donor plant) to the receiver compartment (RC). N transferred corresponds to the amount of rhizodeposited N taken up by *B. napus* L. (receiver plant). N transfer (%) corresponds to the proportion of total N transferred from *T. incarnatum* L. to *B. napus*. Proportion of N derived from the transfer (%Ndft) corresponds to the proportion of total N in *B. napus* L. derived from *T. incarnatum*. Values correspond to the mean ± SE for n = 3. For each parameter, data obtained from +Si plants were compared to −Si plants (control) using Student’s *t*-test. For each parameter, different letters indicate significant differences (*p* < 0.05) between the control (−Si) and +Si plants.

	N Rhizodeposited in the RC (mg)	N Transferred to *B. napus* (mg)	N Transfer (%)	Ndft (%)
**−** **Si**	70.9^a^ ± 7.6	5.7^a^ ± 0.3	0.42^a^ ± 0.03	4.2^a^ ± 0.8
**+Si**	99.8^b^ ± 7.3	23.3^b^ ± 3.8	1.60^b^ ± 0.1	12.2^b^ ± 1.3

**Figure 3:**
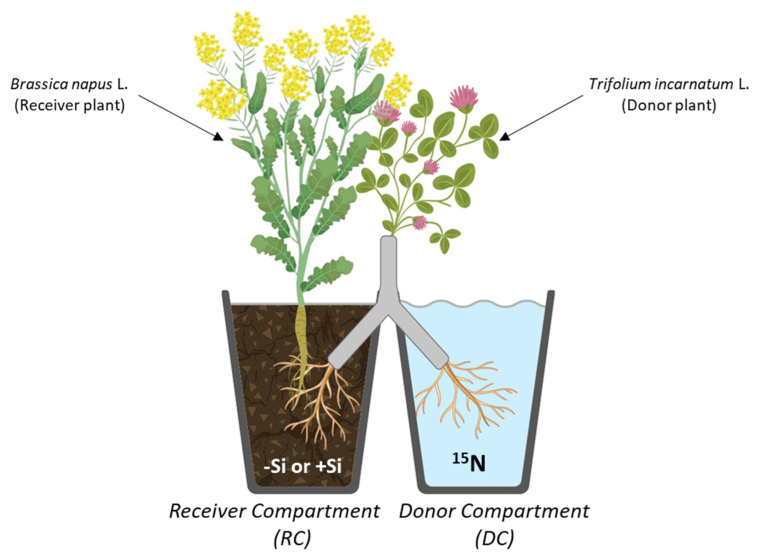
Experimental design. The receiver compartment (RC) contains soil treated (+Si; 1.7 mM) or not (−Si) with Si. The donor compartment contains nutritive solution enriched with K^15^NO_3_ (1 mM, ^15^N excess of 0.5%). *Trifolium incarnatum* L. is the donor plant, with half of its root system growing in DC and the other half growing in RC. *Brassica napus* L. is the receiver plant growing in RC.

## 3. Discussion

As part of the current agro-ecological transition, crop associations are considered an effective cropping practice for reducing the doses of nitrogen fertilizers required to ensure the growth and yield of many crops, such as *B. napus* L. [30,31]. For example, an earlier study performed on different *B. napus* L.-legume associations showed that *T. incarnatum* L. is highly effective at enhancing rhizodeposited N-compounds, which increase the pool of available N in the soil [17]. The same study also reported that a significant part of these N-compounds are taken up by *B. napus* L. and promote its growth [17]. In addition, several studies have reported a beneficial effect of Si supply on the N nutrition and growth of both of these plant species cultivated in monoculture [26,29]. However, to date, the impact of Si supply on the performance of the *B. napus* L.-*T. incarnatum* L. crop association has never been studied. Therefore, in the present study, the use of a split root system combined with ^15^N labeling was performed to assess the effect of Si supply on N transfer from *T. incarnatum* to *B. napus* L. when these two species are cultivated in association. In particular, this work focused on the rhizodeposition of N compounds by *T. incarnatum* L. and their transfer to *B. napus* L., as well as their distribution in the different compartments of *B. napus* L.

This study showed that *T. incarnatum* L. rhizodeposits N, and that part of this N is transferred to *B. napus* L., irrespective of the Si supply (Table 3 and Figure 2). This result is in line with previous studies that reported that in *B. napus* L. -legume associations, N compounds rhizodeposited by legumes are transferred to *B. napus* L. [8,17,32]. Interestingly, for the first time, our study showed that Si supply highly increases the capacity of N rhizodeposition by *T. incarnatum* L. (Table 3 and Figure 2) and leads to an amount of rhizodeposited N in soil enhanced by more than 40%. The effect of Si on the increased rhizodeposition of N by *T. incarnatum* L. might be a consequence of the positive effect of Si on nodulation and the N fixation capacity previously demonstrated in legumes and in particular in *T. incarnatum* L. [28,33]. The N_2_-fixing capacities of *T. incarnatum* L. could also be enhanced by the N depletion in the soil, which would be favored by the increase in the N uptake capacity of *B. napus* L. in response to Si supply [15,17,23]. The hypothesis that improving the N_2_-fixing of *T. incarnatum* L. could increase the N rhizodeposition capacity is corroborated by previous studies, indicating that when associated with *B. napus* L., the legume plants with the best N_2_-fixing capacity are also those with the greatest root N exudation capacity [15,17].

This study showed that Si supply resulted in a 4-fold increase in the N transfer from *T. incarnatum* L. to *B. napus* L. (Table 3 and Figure 2). This improved N transfer was due to an increase in the uptake of N-rhizodeposited compounds by *B. napus* L. (Figure 2). This result is in line with a previous study that reported that, in *B. napus* L. grown under field conditions, Si supplementation increases the efficiency of the uptake of available nitrogen from the soil solution [26]. Furthermore, this process may be due to the Si induction of genes encoding root N transporters (such as *BnaNRT1.1* and *BnaNRT1.2*), as previously observed in N-deficient *B. napus* L. [23]. This increase may also be favored by the rhizodeposition of N compounds that are more easily taken up by *B. napus* L., such as ammonium. This is in agreement with earlier work reporting that ammonium is the main N compound exuded by *Trifolium repens* L. cultivated under N deficiency [34]. Furthermore, in RC, it cannot be excluded that *T. incarnatum* L. deposits organic N-compounds, the mineralization of which would be enhanced by the addition of Si. This hypothesis is in line with a previous study performed on rice, which reported that Si promotes the mineralization of N compounds by stimulating the growth of soil microorganisms involved in this process [35].

Finally, this better N transfer to *B. napus* L. in response to Si supply led to (i) a decrease in the proportion of residual rhizodeposited N in +Si RC soil (76% compared with 92% in −Si RC soil) (Figure 2) and (ii) an increase in the proportion of N within *B. napus* L. that derived from *T. incarnatum* L. (Ndft) (Table 3). Indeed, the proportion of transferred N increased from 4.2% to 12.2% of the total N in −Si and +Si *B. napus* L., respectively (Table 3). As presented in Figure 2, this excess transferred N is preferentially allocated to pods of +Si *B. napus* L. (17% vs. only 1.5% in pods of −Si plants). Interestingly, this preferential allocation of N to pods led to a significant increase in their biomass, which reached 4.6 g in +Si *versus* only 2.6 g in −Si *B. napus* L. (Figure 1). These results suggest that Si supply can improve the yield and quality of harvested products in *B. napus* L. This is in agreement with recent work showing that Si increases the number of seeds and the proportion of N allocated to them in several plant species, such as rice and maize [35,36]. However, these results obtained under greenhouse conditions require validation under field conditions with *B. napus* and *T. incarnatum* cultivated in association and treated with Si fertilizers and reduced N inputs to consider future improvements in agricultural practices for *B. napus* crops.

## 4. Materials and Methods

### 4.1. Plant Growth Conditions and Experimental Design

This experiment was performed with seeds of *T. incarnatum* L. (var. Cegalo) and *B. napus* L. (var. Mosaik) provided by the Jouffray-Drillaud Cérience (Cissé, France) and KWS Momon (Mons-en-Pévèl, France) seed companies, respectively. Seeds were germinated on perlite over deionized water during four days in the dark and then transferred to natural light conditions for one week until the first leaf emerged. The seedlings were supplied for one and a half weeks with nutrient solution containing the following chemical compounds (Merck, Darmstadt, Deutschland): KNO_3_ (1 mM), KH_2_PO_4_ (0.25 mM), KCl (1 mM), CaCl_2_ (3 mM), MgSO_4_ (0.5 mM), EDTA-2NaFe (0.2 mM), H_3_BO_3_ (14 µM), MnSO_4_ (5 µM), ZnSO_4_ (3 µM), CuSO_4_ (0.7 µM), (NH_4_)_6_Mo_7_O_24_ (0.7 µM), and CoCl_2_ (0.1 µM). Then, both seedling species were transplanted into hydroponic tanks (20 L, 10 plants per tank) containing the nutrient solution described above, and the roots of *T. incarnatum* L. were inoculated with *Rhizobium leguminosarum* bv *trifolii* (strain T354, MSDJ1056, [18,37]). After two weeks, one plant of *B. napus* L. was transplanted into a pot (4 L), named the “receiver compartment” (RC), containing 5.5 kg of a mix of silty-clay soil and sand (*v*:*v* 1/3). For *T. incarnatum* L., roots were separated into two equal parts using a Y split-root design: one half of the roots was placed in the “donor compartment” (DC) containing 4 L of the nutrient solution previously described, labelled with K^15^NO_3_ (isotopic excess = 0.5%), and the remaining half of the roots was placed in the “receiver compartment” (RC) containing *B. napus* L. (Figure 3). The nutrient solution in the DC was continuously aerated with a compressed air bubbling system, and its pH was monitored each day and adjusted if necessary to 5.8 ± 0.2. The nutrient solution of the DC was renewed every three days and inoculated with 10 mL of medium containing *Rhizobium leguminosarum* bv *trifolii*. The RC was watered daily with 60 mL of the nutrient solution described above supplemented with 1.7 mM silicon (Si as sodium metasilicate: Na_2_SiO_3_) for +Si plants or 3.4 mM NaCl (to compensate for the Na supplied by the Si treatment) for −Si (control) plants.

Throughout the experiment, plants were grown in controlled conditions in a greenhouse (Caen, Normandy, France). Natural light was supplemented by high-pressure sodium lamps (Master GreenPower T400W, Philips, Villeneuve-Saint-Georges, France) with photosynthetically active radiation of 450 μmol photons·m^−2^·s^−1^ at canopy height. Plants were harvested after 10 weeks of Si supply and ^15^N labeling (from 23 March to 4 June 2024). The period of 10 weeks was chosen to enable the *T. incarnatum* to develop sufficiently to maximize N_2_ fixation, rhizodeposition, and the transfer of ^15^N-compounds to *B. napus*. In addition, this period allows us also to obtain more than 50% of siliques with final size in *B. napus* (stage BBCH75 [38]), and thus evaluate the allocation of N-rhizodeposited in the different plant compartments. Shoots and roots were separated and weighed, and an aliquot of each was dried at 60 °C for 72 h. After dry weight determination, samples were ground to perform the elemental analyses.

### 4.2. Determination of N and ^15^N Amount

To determine the total N and ^15^N concentrations, 1.5 mg of each dried powder was precisely weighed and put into tin capsules. Samples were analyzed with a continuous flow isotope ratio mass spectrometer (IRMS, Horizon, NU Instruments, Wrexham, UK) linked to a C/N/S analyzer (EA3000, Euro Vector, Milan, Italy). The total N amount (N_tot_) in each plant compartment was calculated as:

(1) $$N_{tot}=\frac{\%N\times DW}{100}\;$$

The amount of ^15^N (^15^N) in each compartment was determined by the following:

(2) N15=%N15 excessof compartment×Ntot%N15 excesslabelled of nutrient solution  

With: ^15^N excess _labelled of nutrient solution_ = 0.5%

To estimate the total N transfer from the donor plant (*T. incarnatum* L.) to the receiver plant (*B. napus* L.), it was assumed that ^15^N and ^14^N were transferred in equal proportions. According to Ledgard et al. [39], the ratio between ^15^N in the receiver and the total ^15^N (in both the receiver and the donor plants) was used to calculate the percentage of total N transferred from the donor to the receiver (*B. napus* L. (*%_N transfer_*) or soil (*%_residual N rhizodeposited_*).

(3) %N transfer(or %residual N rhizodeposited)=N15 amountrapeseed (or soil)N15 amountrapeseed(or soil)+N15amountdonor×100

Then, the total amount of N transferred (mg N) from the donor to the receiver plant (*N_transfer_*) or the total amount of residual N rhizodeposited (ResNrhizo.; mg N) from the donor to the soil was calculated as:

(4) $$N_{transfer}(or\;ResNrhizo)=\frac{{\%}_{N\;transfer}\left(or\;{\%}_{residual\;N\;rhizodeposited}\right)\times N_{tot\;donor}}{100}$$

Finally, the percentage of N derived from transfer (%Ndft) in *B. napus* L., i.e., the proportion of total N in *B. napus* L. derived from *T. incarnatum*, was calculated as:

(5) $$\%Ndft=\frac{N_{transfer}\times 100}{N_{tot\;receiver}}$$

### 4.3. Statistical Analysis

The experiment was performed with three independent biological replicates. All data are represented as the mean ± S.E. (n = 3). Statistical analyses were performed using R software (version 4.2.0: R Core Team, 2022): significant differences between control plants (−Si) and +Si plants were determined using Student’s *t*-test (*p* < 0.05).

## 5. Conclusions

For the first time, this study highlights the beneficial effect of Si supply on the agronomic performance of the *B. napus* L. cultivated in association with the legume plant *T. incarnatum* L. through the promotion of the rhizodeposition of N compounds by *T. incarnatum* L. (more than 40%) and the increased uptake of these compounds by *B. napus.* In addition, the positive effect of Si on the growth of *B. napus* L. in association with *T. incarnatum* L. opens up new avenues for reducing N inputs. Thus, the combination of Si-based fertilizers and their association with *T. incarnatum* could improve the environmental balance of *B. napus* L. crops by increasing their N use efficiency, which is one of the main challenges facing the oilseed industry. In the context of the agro-ecological transition, future experiments in field conditions will be needed to test whether the application of Si-based fertilizers to *B. napus* L. grown in association with *T. incarnatum* L. can ensure optimal yields under lower N inputs.

## Data Availability

The original contributions presented in this study are included in the article. Further inquiries can be directed to the corresponding author.
